# Acknowledgement to Reviewers of *Antibodies* in 2018

**DOI:** 10.3390/antib8010004

**Published:** 2019-01-01

**Authors:** Antibodies Editorial Office

**Affiliations:** MDPI AG, St. Alban-Anlage 66, 4052 Basel, Switzerland

Peer review is an essential part of the publication process, ensuring that *Antibodies* maintains high quality standards for its published papers. In 2018, a total of 40 papers were published in the journal. Thanks to the cooperation of our reviewers, the median time to first decision was 18 days, and the median time to publication was 42 days. The editors would like to express their sincere gratitude to the following reviewers for their time and dedication during 2018:
Adolf-Bryfogle, JaredJiang, LiuweiAsano, RyutaroJin, JiefuBandi, Venu GopalKim, Kristine M.Blasi, JuanKnapp, BernhardBowen, AnthonyKolodych, SergiiBoyd, AndrewKontermann, RolandCaaveiro, JoseKuhlman, BrianCavallari, MarcoKwon, Myung-HeeChalla, DilipLerchen, Hans-GeorgChen, Wei-YuLeusen, Jeanette H.Cheng, HailiLi, JinpingChoi, Bryan D.Li, WeiChoudhari, ShyamalLiu, XiangleiChowdhury, RatulLub-de Hooge, MarjolijnChudasama, VijayLyu, YuanzhiChung, JunhoMaggi, EnricoConroy, PaulMalik, RavinderDe Kruif, JohnMallipeddi, Prema LathaDixit, SurjitMassa, SamEisenreich, AndreasMatikonda, SiddharthEpstein, Alan L.Mayer, AndreasEscobar-Cabrera, EricMazor, OhadGogulamudi, Venkateswara ReddyMølhøj, MichaelGoldman, Ellen R.Moricoli, DiegoGonzalez, DeyarinaMukherjee, SomnathGorovits, BorisMuyldermans, SergeGreiff, VictorOpaliński, ŁukaszHouse, ImranOwen, ShawnJakobsson, EricOzen, Mehmet OzgunPacios, Luis F.Storti, GiuseppePiatkevich, KirylSun, WeiPluen, AlainSun, YingjiePrat, MariaTalukder, PoulamiRasche, LeoTanha, JamshidRissiek, BjoernTeicher, Beverly A.Ronsard, LaranceVaidyanathan, BharatRossotti, Martin A.Valko, KlaraRovero, PaoloVance, DavidSchmidt, FlorianVan Leeuwen, F. W. B. Schrum, AdamVattepu, RaviSherbenou, Daniel W.Vieth, MichalShim, HyunboWang, JunjieShirouzu, MikakoWang, Lai-XiShityakov, SergeyWheatley, AdamSidhu, SachdevWiddison, Wayne C.Sil, PayelWozniak-Knopp, GordanaStasevich, Timothy J.Zhao, Jun

